# Fast field echo resembling CT using restricted echo-spacing (FRACTURE) MR sequence can provide craniocervical region images comparable to a CT in dogs

**DOI:** 10.3389/fbioe.2024.1297675

**Published:** 2024-02-27

**Authors:** Dongjae Lee, Eunjee Kim, Hyeonjae Woo, Chang-Yeop Jeon, Junghee Yoon, Jihye Choi

**Affiliations:** ^1^ Department of Veterinary Medical Imaging, College of Veterinary Medicine, Seoul National University, Seoul, Republic of Korea; ^2^ National Primate Research Center, Korea Research Institute of Bioscience and Biotechnology, Cheongju, Republic of Korea

**Keywords:** bone, canine, CT-like, MRI, vertebra

## Abstract

Magnetic resonance imaging (MRI) is essential for evaluating cerebellar compression in patients with craniocervical junction abnormalities (CJA). However, it is limited in depicting cortical bone because of its short T2 relaxation times, low proton density, and organized structure. Fast field echo resembling a computed tomography (CT) scan using restricted echo-spacing (FRACTURE) MRI, is a new technique that offers CT-like bone contrast without radiation. This study aimed to assess the feasibility of using FRACTURE MRI for craniocervical junction (CCJ) assessment compared with CT and conventional MRI, potentially reducing the need for multiple scans and radiation exposure, and simplifying procedures in veterinary medicine. CT and MRI of the CCJ were obtained from five healthy beagles. MRI was performed using three-dimensional (3D) T1-weighted, T2-weighted, proton density-weighted (PDW), single echo-FRACTURE (sFRACTURE), and multiple echo-FRACTURE (mFRACTURE) sequences. For qualitative assessment, cortical delineation, trabecular bone visibility, joint space visibility, vertebral canal definition, overall quality, and artifacts were evaluated for each sequence. The geometrical accuracy, signal-to-noise ratio (SNR), and contrast-to-noise ratio (CNR) were quantified. Both sFRACTURE and CT images provided significantly higher scores for cortical delineation and trabecular bone visibility than conventional MRI. Joint space visibility and vertebral canal definition were similar to those observed on CT images, regardless of the MR sequence. In the quantitative assessment, the distances measured on T2-weighted images differed significantly from those measured on CT. There were no significant differences between the distances taken using T1-weighted, PD-weighted, sFRACTURE, mFRACTURE and those taken using CT. T1-weighted and sFRACTURE had a higher SNR for trabecular bone than CT. The CNR between the cortical bone and muscle was high on CT and FRACTURE images. However, the CNR between the cortical and trabecular bones was low in mFRACTURE. Similar to CT, FRACTURE sequences showed higher cortical delineation and trabecular bone visibility than T2-weighted, T1-weighted, and PDW CCJ sequences. In particular, sFRACTURE provided a high signal-to-noise ratio (SNR) of the trabecular bone and a high CNR between the cortical bone and muscle and between the cortical and trabecular bones. FRACTURE sequences can complement conventional MR sequences for bone assessment of the CCJ in dogs.

## 1 Introduction

Abnormalities of the craniocervical junction (CJA) are common in veterinary medicine. CJA is a general term used to describe malformations of the craniocervical region (CCJ), such as Chiari-like malformations, atlantooccipital instability, atlantoaxial instability, occipitoatlantoaxial malformations, atlantooccipital overlapping, and dens abnormalities in small-breed dogs. Magnetic resonance imaging (MRI) provides excellent assessment of soft tissues and medullary bone without ionizing radiation. Therefore, MRI is essential for evaluating cerebellar compression in patients with CJA. However, in diagnosing CJA, it is often difficult to determine which bone structures are involved in the abnormality and which areas are affected secondary to CJA. MRI is limited in depicting the cortical (including the subchondral) bone because of its very short T2 relaxation times, low proton density, and organized structure (Marino et al., 2012). Computed tomography (CT) is often performed in addition to MRI to determine the bone structures that cause neural tissue compression in human patients with CJAs and in veterinary medicine, because CT can visualize the bone structures in detail with high resolution (Goel et al., 1998; Botelho et al., 2007; Cerda-Gonzalez et al., 2009a; Marino et al., 2012; Stalin et al., 2015; Kiviranta et al., 2017; Planchamp et al., 2022). In addition, three-dimensional (3D) isotropic CT allows multiplanar and volumetric reconstruction to help depict bone and joint structures (Kwon et al., 2005; Griffith et al., 2007; Bishop et al., 2013; Chong et al., 2021). Despite these advantages, patient exposure to ionizing radiation is a primary concern in CT in humans. Moreover, additional CT scans after MRI require the complex process of moving the patient from the MR machine to the CT room while maintaining the correct position of the patient for the CT scans. Unlike humans, animals generally require anesthesia for MRI and CT scans. This process requires a long anesthesia time, and many personnel, which incur high examination costs.

With the improvement of MRI techniques, new MR sequences that can depict the cortical bone and provide CT-like bone contrast (CLBC) have been introduced in human medicine (Chong et al., 2021). There are various 3D MRI approaches for generating CLBC images, including the ultrashort echo time gradient echo (UTE) (Chang et al., 2015), zero echo time (ZTE) (Kwon et al., 2005; Griffith et al., 2007; Bishop et al., 2013), T1-weighted gradient-recalled echo(GRE) pulse sequences (Dremmen et al., 2017; Gersing et al., 2019), susceptibility-weighted imaging (SWI) pulse sequences (Böker et al., 2018; Böker et al., 2019; Böker et al., 2020; Haller et al., 2021), and deep learning methods (Han, 2017).

Fast field echo resembling a CT scan using restricted echo-spacing (FRACTURE) is a Cartesian 3D GRE-pulse sequence that generates CLBC 3D MRI with high spatial resolution. FRACTURE is a high-resolution 3D gradient-echo-based technique that uses multiple echoes with constant echo spacing and post-processing subtraction to provide CT-like image contrast. 3D gradient echo–based sequences have been shown to provide high bony contrast by providing images with low-signal bone contours compared to the surrounding high-signal fatty bone marrow and soft tissue. After acquisition, two additional post-processing steps are performed to produce images with more CT-like contrast. The first post-processing step consists of a summation of the magnitude of all the echoes. This increases the signal-to-noise ratio, which is inherently low due to the high resolution and receiver bandwidth. After summation, the images from the last echo are subtracted from the summed images. This inverts the grey scale and gives the bone CT-like contrast. The advantages of 3D Cartesian gradient echo-based sequences are that they are commonly available on MRI scanners from all vendors, have field strengths, permit high-spatial-resolution imaging, and use small flip angles for lower specific absorption rates (Johnson et al., 2021).

Based on previous human studies, we anticipated that CLBC 3D MR sequence could complement bone structure assessment after soft tissue evaluation using a FRACTURE MR scan instead of an additional CT scan. The use of CLBC images would simplify the examination of CJAs and reduce costs for patients for whom preoperative planning with MRI and CT is typically required (Chong et al., 2021). However, to the best of our knowledge, there have been no published clinical studies on CCJ evaluation using FRACTURE sequence MRI in dogs. We hypothesized that FRACTURE MR can provide similar resolution images to CT images of the cortical bone of the CCJ in dogs. This study aimed to describe the MR features of the CCJ in normal dogs using 3.0-T MRI and assess the feasibility of FRACTURE sequences for CCJ evaluation by comparing them with CT and conventional MR images in dogs.

## 2 Materials and methods

### 2.1 Selection and description of subjects

In this prospective method comparison pilot study, five clinically healthy purpose-bred beagles (three neutered males and two intact females) were used (mean body weight: 13 kg (range: 9.9–15.5 kg); mean age: 3 years (range: 2–5 years). The sample size was determined using convenience sampling. The dogs were housed individually and provided commercial dry food and tap water *ad libitum*. All dogs were clinically healthy based on a physical examination, complete blood count, serum biochemistry, electrolytes, cranial nerve examination, and thoracic and abdominal radiography. The CCJs of all dogs were determined to be normal based on CT findings. All procedures performed in this study were approved by the Seoul National University Institutional Animal Care and Use Committee (SNU IACUC-230726-2).

### 2.2 Anesthesia and schedule

In each dog, MRI and CT scans were performed in random order at 7–9-day intervals using the same anesthesia procedure. After each dog had been fasted for 12 h, a 24-gauge catheter was placed in the cephalic vein to induce anesthesia. After premedication with medetomidine (0.01 mg/kg IM, Domitor, Zoetis, Finland), anesthesia was induced with alfaxalone (2.0 m/kg IV, Alfaxan, Jurox Pty Ltd., Australia) and maintained with isoflurane (Ifran, Hana Pharm, South Korea) in oxygen. For CT and MRI, noninvasive blood pressure, oxygen saturation, heart rate, respiratory rate, and body temperature were monitored during the induction and maintenance of anesthesia.

The general condition and anesthesia-related side effects, such as respiratory signs, vomiting, depression, and anorexia, of each dog were monitored for 5 days postoperatively.

### 2.3 MRI examinations

In all dogs, MR of the head and neck was performed using a 3.0-T MRI scanner (Achieva, Philips Healthcare, Netherlands) with a 32-channel cardiac coil. All dogs were positioned in sternal recumbency with both forelegs pulled back and head-first. T1-weighted (T1W) images were obtained as localizers in three orthogonal planes using 3D turbo field-echo (TFE) scans. The field of view (FOV) was set to include the CCJ from the olfactory bulb to the axial level in all planes (Deininger-Czermak et al., 2021). The following were then obtained from each dog: 1) T2-weighted (T2W) images using 3D turbo spin-echo (TSE) sequences, 2) T1-weighted (T1W) images using 3D TSE sequences, 3) proton density-weighted (PDW) images using 3D TSE sequences, 4) 3D fast-field echo sequences using sFRACTURE, and 5) 3D fast-field echo sequences using mFRACTURE. In this study, mFRACTURE was created by summation of four in-phase echo images. On the other hand, sFRACTURE used only one in-phase echo image to obtain an unsummated image with a lower signal to noise than mFRACTURE. Table 1 shows the parameters and their sequences. In 3D acquisition sequences, sagittal images were acquired, and transverse and dorsal images were reconstructed. The total scan and image acquisition times for each sequence were measured automatically (Deininger-Czermak et al., 2021).

**TABLE 1:** MRI parameters and sequences.

Parameter	MR sequences				
	3D FRACTURE multi-echo	3D FRACTURE single-echo	3D PDW	3D T1W	3D T2W
Number of In phase echo	4	1			
TR (ms)	16	5.4	1,100	350	1,500
TE (ms)	In phase (2.3) Echo-spacing (2.3)	In phase (2.3)	34	19	100
Flip angle	12	8	90	90	90
Slice interval	0.6	0.6	0.6	0.6	0.6
FOV (mm)	160 × 160 × 60	160 × 160 × 60	160 × 160 × 60	160 × 160 × 60	160 × 160 × 60
Matrix	268 × 266	268 × 266	268 × 266	268 × 266	268 × 235
Reconstruction Matrix	320	288	320	320	320
NEX	1	1	1	1	1
Acquisition time (min:sec)	7:01	4:51	12:57	11:55	13:31

MR, magnetic resonance image; CT, computed tomography; mFRACTURE, Fast field Echo Resembling a CT Using Restricted Echo-spacing- multiple echoes; sFRACTURE, Fast field Echo Resembling a CT Using Restricted Echo-spacing- single echo; PDW, Proton density-weighted; T2W, T2-weighted; and T1W, T1-weighted image; TR, repeated time; TE, echo time; FOV, field of view; NEX, number of excitation.

### 2.4 CT examinations

After placing the dog in sternal recumbency, a CT scan of the CCJ was performed using a 160 multi-slice CT scanner (Aquilion Lightning 160, Canon Medical Systems, Japan) with the following parameters; 120 kVp with a tube current of 200 mA, slice thickness of 0.5 mm, and tube rotation time of 1.0 s. The image dataset was reconstructed at a slice thickness of 0.6 mm and an increment of 0.4 mm, using a medium-frequency algorithm for soft tissue and a high-frequency algorithm for bone structures in the transverse and sagittal planes. The FOV was set to include the region from the nose to the cervical vertebrae. A maximum FOV of 200 × 200 mm^2^ with a matrix of 512 × 512 mm^2^ was used.

### 2.5 Image analyses

All MR and CT images were sent to a picture archiving and communication system (Infinitt PACS; Infinitt Healthcare, Seoul, South Korea). Qualitative and quantitative assessments of MRI and CT images were performed individually by two observers (D.J.L. and E.J.K.), each with 1–2 years of radiology experience, in random order under the supervision of a radiologist (J.H.C.) at the Korean College of Veterinary Medical Imaging. All CT images were evaluated using a bone window with a window level of 500 Hounsfield Units (HU) and a window width of 2000 HU.

**FIGURE 1:**
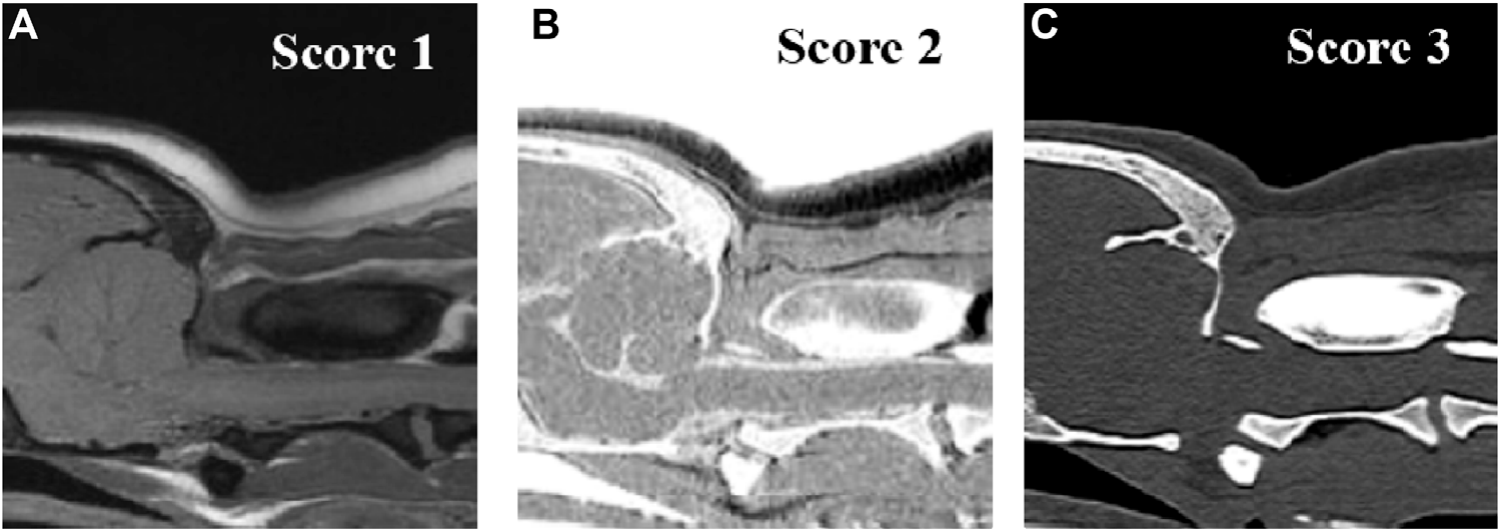
Qualitative evaluation of the image qualities on T1-weighted **(A)**, single echo-FRACTURE **(B)**, CT **(C)** images of craniocervical region in a beagle. 1 = Artifacts present, affecting the images; 2 = Artifacts were present but minimal, not affecting the images; and 3 = Artifacts were absent.

**FIGURE 2:**
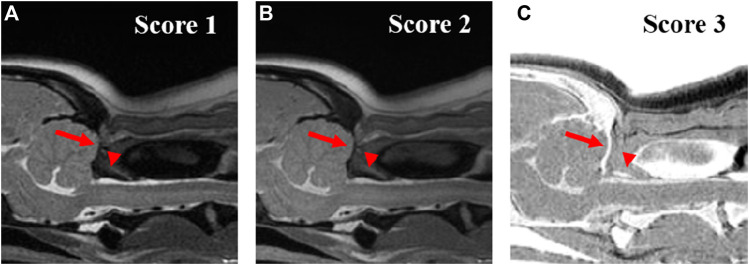
Qualitative evaluation of the visuality of the cortical delineation of occipital region (arrow) and dorsal arch of atlas (arrow-head) on T2-weighted **(A)**, Proton density-weighted **(B)**, single echo-FRACTURE **(C)** images of the craniocervical region in a beagle. 1 = The border of the cortical bone was too blurry to be distinguished from the surrounding soft tissue; 2 = Most cortical bones were well distinguished, but there were some blurry parts; and 3 = The cortical bone was clearly distinguishable from the surrounding soft tissue.

**FIGURE 3:**
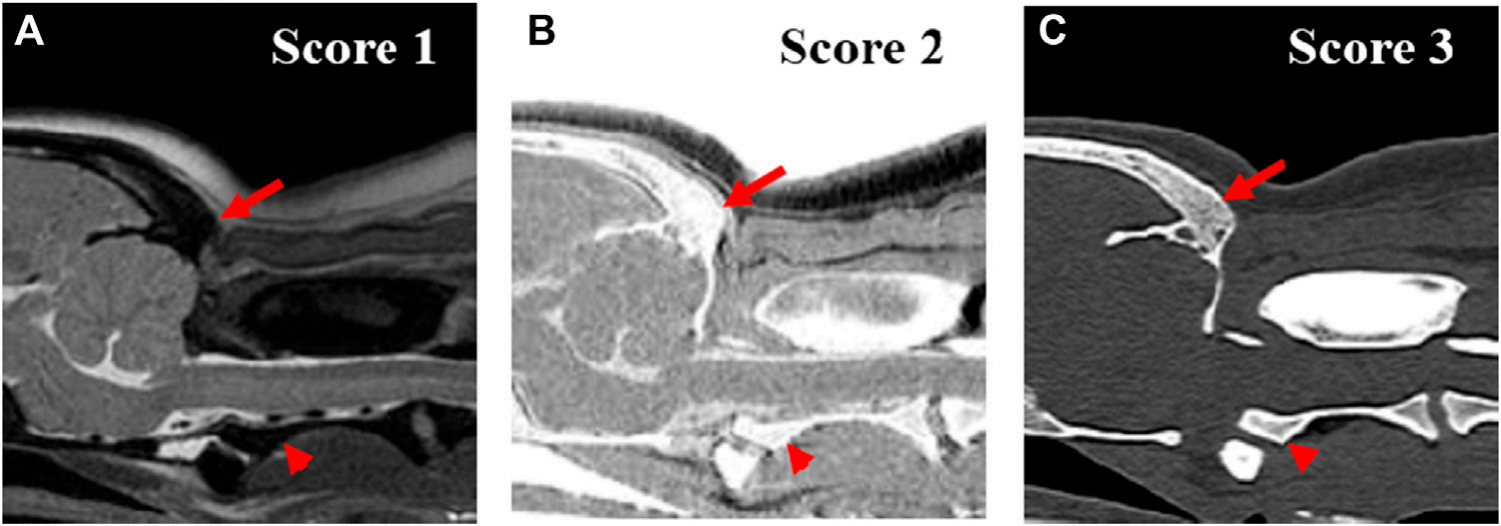
Qualitative evaluation of the clarity of trabecular bone of occipital region (arrow) and axis (arrow head) on T2-weighted **(A)**, single echo-FRACTURE **(B)**, CT **(C)** images of craniocervical region in a beagle. 1 = The trabecular bone appeared homogeneous overall, and there were no visible trabecular patterns; 2 = The trabecular pattern was visible but not clear; 3 = The trabecular and cortical bones were well distinguished, and the trabecular pattern was visible.

Images were assessed at three evaluation sites: the occipital bone, the first cervical vertebra (C1), and the second cervical vertebra (C2). For image assessment, both CT images of the bone window and MRI images were interpreted by scrolling through all images. For the qualitative assessment, the image quality and artifacts of the overall images (Figure 1), visualization of the cortical delineation (Figure 2), clarity of the trabecular bone (Figure 3), conspicuity of the joint spaces, and delineation of the vertebral canal of the evaluation sites were evaluated. Only the delineation of the vertebral canal image was evaluated in the transverse plane, and all others were evaluated in the sagittal plane. The main evaluation site of the occipital bone was the occiput; both the dorsal and ventral arches were evaluated in C1, and the ventral body was the main evaluation site in C2. For image quality, motion, partial volume, and chemical shift artifacts were evaluated separately using a three-point scale: 1 = Artifacts present, affecting the images; 2 = Artifacts were present but minimal, not affecting the images; and 3 = Artifacts were absent. Visualization of cortical delineation was assessed using a three-point scale based on whether the cortical bone and surrounding soft tissue were well distinguished. 1 = The border of the cortical bone was too blurry to be distinguished from the surrounding soft tissue; 2 = Most cortical bones were well distinguished, but there were some blurry parts; 3 = The cortical bone was clearly distinguishable from the surrounding soft tissue. The clarity of the trabecular bone was assessed using a three-point scale based on its differentiation from the cortical bone and visualization of trabecular patterns. 1 = The trabecular bone appeared homogeneous overall, and there were no visible trabecular patterns; 2 = The trabecular pattern was visible but not clear; 3 = The trabecular and cortical bones were well distinguished, and the trabecular pattern was visible. The conspicuity of the joint spaces was assessed using a three-point scale based on the distinction between the joint space and the articulating bones. 1 = It was difficult to distinguish between the parts that comprised the joint and the boundary of the joint; 2 = The bone and joint space were easily distinguished from each other, but there were ambiguous parts; and 3 = The bones forming the joint and the space between them were clearly visible. Delineation of the vertebral canal was assessed using a three-point scale based on the definition of the spinal cord and bones. 1 = The border between the spinal cord and bone was blurred and an accurate distinction was difficult; 2 = The border was slightly blurry but distinguishable; 3 = The spinal cord and surrounding bones were clearly visible.

**FIGURE 4:**
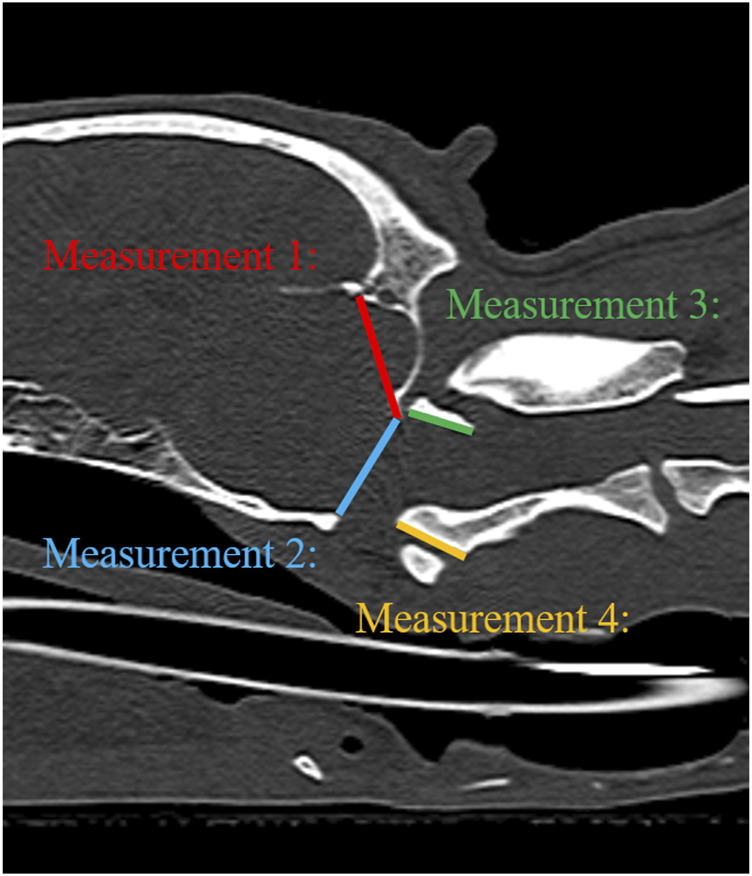
Illustration of measurements 1–4 to evaluate geometric accuracy on CT sagittal images. Measurement 1: Occipital bone length measured from the occipital protuberance to the ventral surface of the occipital bone (opisthion). Measurement 2: Caudal height of the foramen magnum measured from the inside of the basion to the inside of the opisthion. Measurement 3: Length of the dorsal arch of the atlas measured from the ventral line. Measurement 4: Maximum length of the dens measured from the ventral line.

For a quantitative evaluation, the geometric accuracy, signal-to-noise ratio (SNR), and contrast-to-noise ratio (CNR) were measured. To evaluate the geometric accuracy between MRI and CT images, two observers manually measured four distances twice on each randomly aligned MRI and CT image using an electronic digital caliper on the monitor at four sites (Figure 4); 1) occipital bone length-measured from the occipital protuberance to the ventral surface of the occipital bone (opisthion), 2) caudal height of the foramen magnum-measured from the inside of the basion to the inside of the opisthion, 3) length of the dorsal arch of the atlas-measured from the ventral line, and 4) maximum length of the dens measured from the ventral line. All measurements were performed in the sagittal plane and included two sections from the occipital bone and one section each from C1 and C2. Measurements of the occipital bone length, caudal height of the foramen magnum, and length of the dorsal arch of the atlas were taken from the same cross-section as the most aligned atlanto-occipital joint, and the maximum length of the dens was measured from the cross-section with the maximum dens length.

**FIGURE 5:**
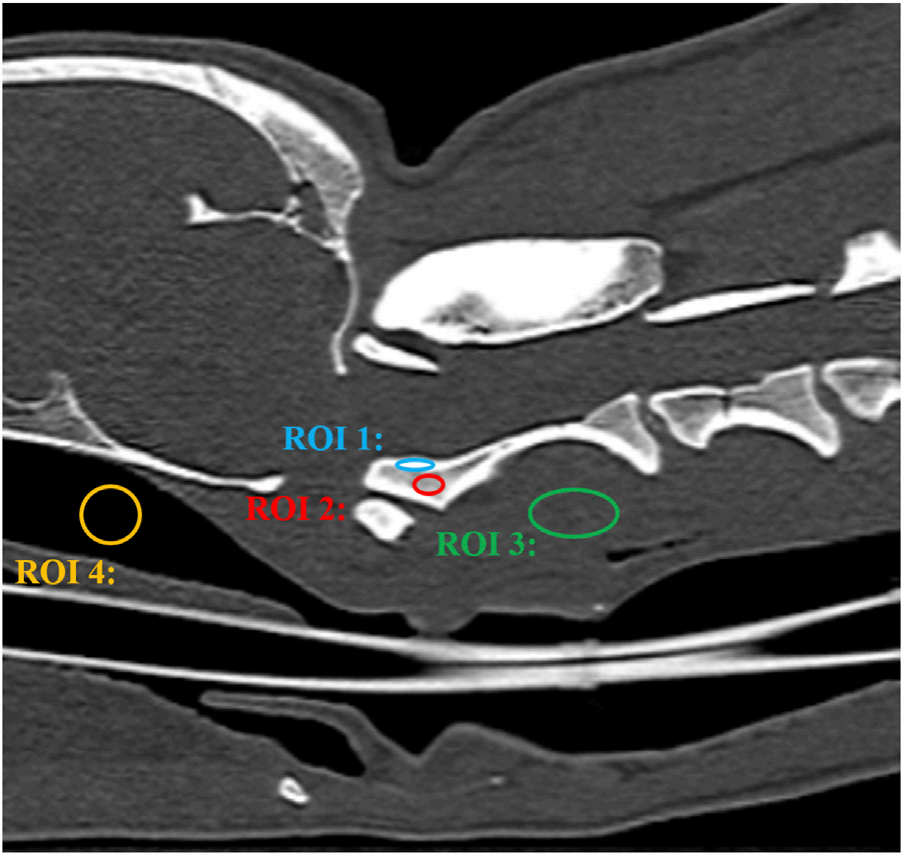
CT image of a craniocervical region in the sagittal plane. The signal intensity of the cortical bone was measured by placing a oval region of interest (ROI) over the cortical bone of axis (ROI 1). The signal intensity of the trabecular bone was measured by placing a oval region of interest (ROI) over the trabecular bone of axis (ROI 2). The signal intensity of the surrounding muscle was measured by placing a circular or oval ROI over the muscle adjacent to the axis (ROI 3). Background noise was measured by placing a circular or oval ROI in the air of the nasopharynx (ROI 4).

The SNR and CNR were calculated to evaluate the quality of MRI and CT images. The SNR of the cortical and trabecular bones, CNR of the cortical and trabecular bones, and CNR of the cortical bone and surrounding muscle (longus colli muscle) were measured in the largest dimensioned slice, where the vertebral body and spinous process of C2 were visible. For this purpose, the signal intensity (SI) of a 5–10 mm^2^ region of interest (ROI) was measured in C2 (cortical bone, trabecular bone) and muscle. The ROI was drawn to be as large as possible, with the exclusion of blood vessels and artifacts. Background noise was measured by placing a circular or oval ROI in the air of the nasopharynx (Figure 5). All measurements were repeated three times, and the average values were used to calculate the SNR and CNR (Usamentiaga et al., 2018). SNR and CNR were calculated as follows:

$$SNRcortical\;bone=\frac{SI\;\left(cortical\;bone\right)}{SD\;\left(air\right)}$$

$$SNRtrabecular\;bone=\frac{SI\;\left(trabecular\;bone\right)}{SD\;\left(air\right)}$$

$$CNRcortical\;bone,muscle=\frac{SI\left(cortical\;bone\right)-SI\left(muscle\right)}{SD\;\left(air\right)}$$

$$CNRcortical\;bone,trabecular\;bone=\frac{SI\left(cortical\;bone\right)-SI\left(trabecular\;bone\right)}{SD\;\left(air\right)}$$

### 2.6 Statistical analyses

Statistical analysis was performed by a statistician (J.Y.P.) using commercially available software (SPSS statistical program, IBM SPSS Statistics 27.0, IBC Corporation, NY). The non-parametric Wald–Wolfowitz test was used to measure the likelihood of measurement of five dogs with the same expression distribution (Wald and Wolfowitz, 1943). Differences in qualitative assessment (image quality and artifacts, visualization of cortical delineation, clarity of the trabecular bone, conspicuity of the joint spaces, and delineation of the vertebral canal), SNR of the cortical bone, SNR of the trabecular bone, CNR of the cortical bone-muscle, CNR of the cortical bone-trabecular bone, and geometrical measurements were analyzed using the Wilcoxon signed-rank test. To assess geometrical accuracy, the limits of agreement between MRI and CT were calculated for each of the four distances measured using Bland-Altman plot. The interrater agreement between the two observers for the MRI and CT images was evaluated using the interclass correlation coefficient (ICC) test (Landis and Koch, 1977): ICC <.4, poor agreement; ICC .41–.6, moderate agreement; ICC .61–.8, good agreement; ICC >.8, excellent agreement. All data are presented as mean ± standard deviation, and *p* < 0.05 was considered significant.

## 3 Results

**FIGURE 6:**
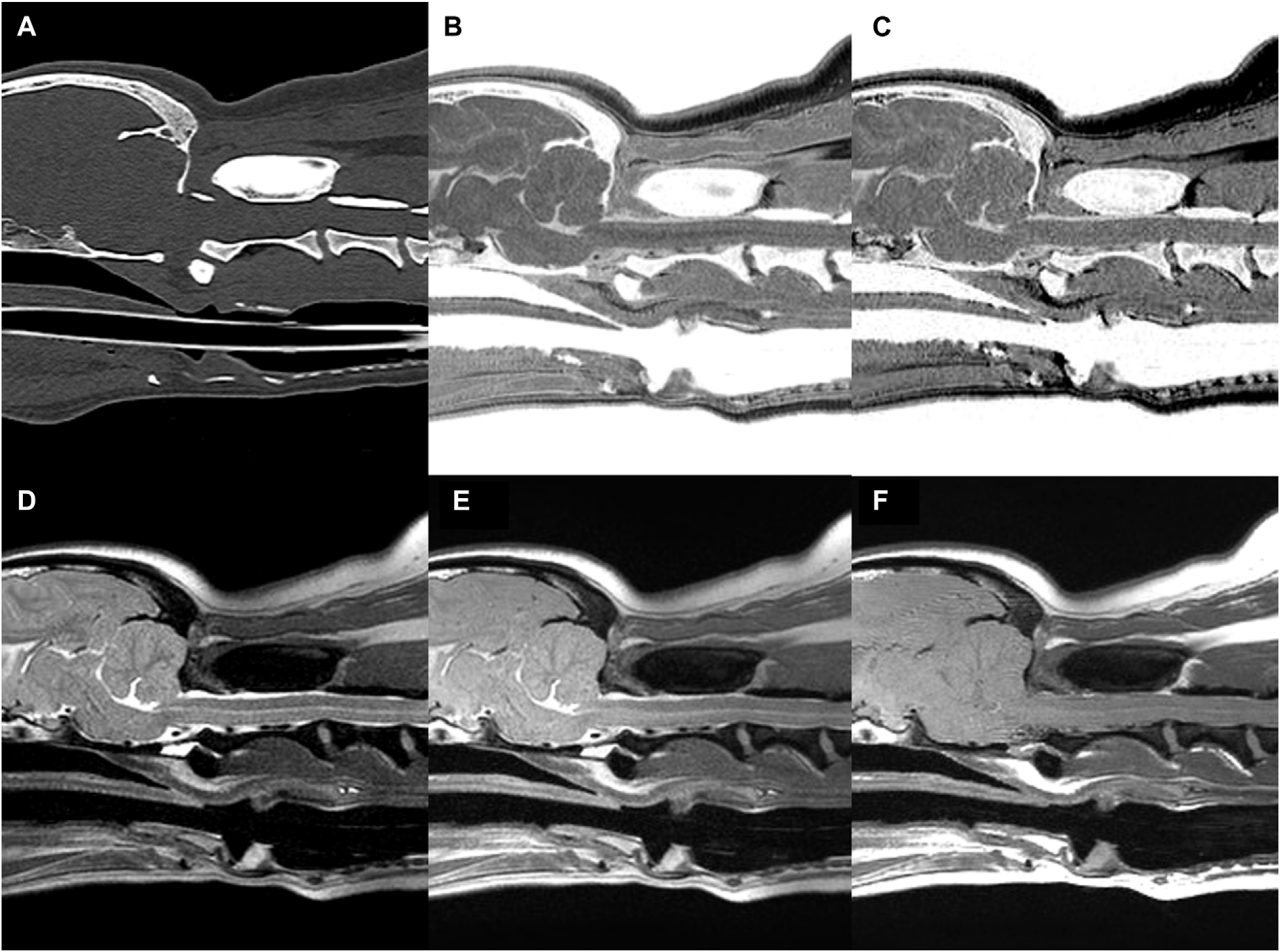
CT **(A)** and MR **(B–F)** images of the craniocervical junction (CCJ) in a beagle. Multiple echo-FRACTURE **(B)** and single echo-FRACTURE **(C)** showed higher cortical delineation and trabecular bone visibility than T2-weighted **(D)**, proton density-weighted **(E)**, and T1-weighted **(F)** images of CCJ, similar to that observed on CT images.

MR and CT images of the CCJ were obtained successfully in all dogs, using FRACTURE sequences, without any complications (Figure 6). The image acquisition times for each MRI sequence are listed in Table 1.

**FIGURE 7:**
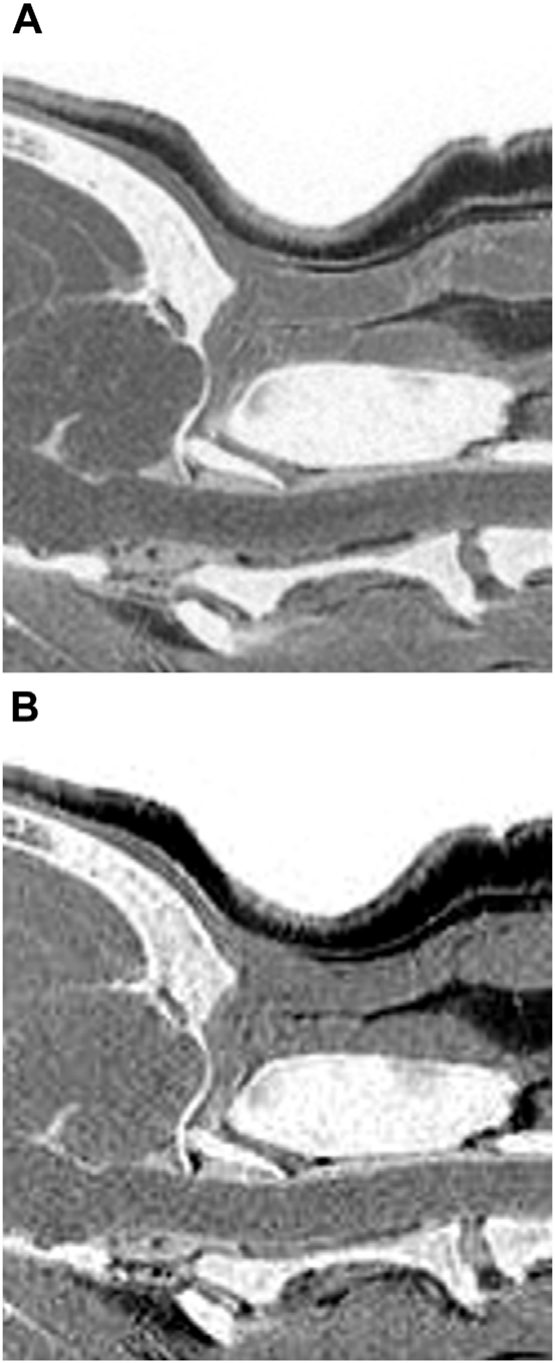
Multiple echo-FRACTURE **(A)** and single echo-FRACTURE **(B)** images of the craniocervical junction (CCJ) in a beagle. Single echo-FRACTURE showed higher trabecular bone visibility than multiple echo-FRACTURE.

**FIGURE 8:**
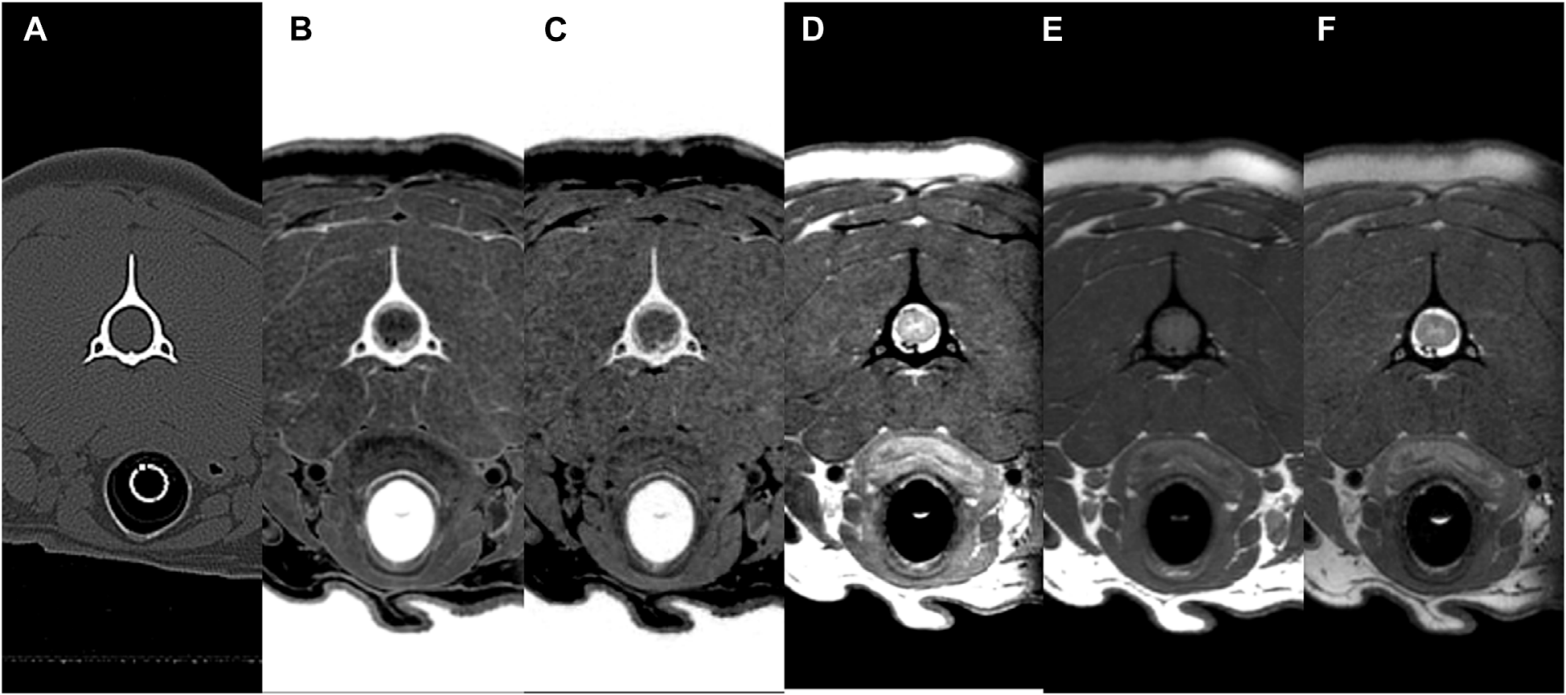
CT **(A)** and MR **(B–F)** transverse images of the vertebral canal at C2 (axis) in a beagle. All sequences (CT, multiple echo-FRACTURE **(B)**, single echo-FRACTURE **(C)**, proton density-weighted **(D)**, T1-weighted **(E)**, and T2-weighted **(F)** had high scores for the evaluation of vertebral canal delineation.

The mean and standard deviation (SD) values for each evaluation factor used in the qualitative evaluation are presented in Table 2. The ICC results for all qualitative evaluation factors were >.8, showing excellent agreement. The image qualities of mFRACTURE and T2W were significantly higher than those of the other sequences, including sFRACTURE. However, T1W had the lowest image quality score, similar to that of PDW. There were no significant differences in visualization of cortical delineation between any of the sequences at the occipital bone and C1 level, whereas sFRACTURE, mFRACTURE, and CT had significantly higher scores than T1W and T2W at C2. The clarity of the trabecular bone had a high score with sFRACTURE and CT at all evaluation sites. In particular, sFRACTURE scored significantly higher than mFRACTURE, PDW, and T2W at the occipital bone (Figure 7). CT also scored significantly higher than all other sequences, except for sFRACTURE. At the C1 level, T2W had the lowest score which was significantly worse than those of sFRACTURE, mFRACTURE, and CT. At the C2 level, CT was not significantly different from sFRACTURE or mFRACTURE. The conspicuity of the joint margin was found to be lower at the level of the occipital bone, with a mean of 1.8 T1W, which was significantly worse than that of all other sequences. At C1, T2W had a mean of 1.8, and scored significantly lower than all sequences except T1W. At C2, sFRACTURE, mFRACTURE, and CT scores were significantly higher than those for PDW, T1W, and T2W. Vertebral canal delineation showed mean scores above 2.0, with no significant difference between sequences at either the C1 or C2 levels (Figure 8). The mean and SD values for the qualitative assessment are shown in Table 2. The ICC results of the qualitative evaluation were all >.8, showing excellent agreement.

**TABLE 2:** Scores of several parameters of magnetic resonance imaging (MRI) and computed tomography (CT).

Item	Level	CT	mFRACTURE	sFRACTURE	PDW	T1W	T2W
Image quality and artifacts		2.80 ± 0.45^a^	2.80 ± 0.45^b,c^	2.20 ± 0.45^d^	2.00 ± 0.71^b,e^	1.00 ± 00^a,c,d,f^	2.20 ± 0.45^e,f^
Visualization of cortical delineation	Occipital	3.00 ± 0.00	3.00 ± 0.00	3.00 ± 0.00	2.60 ± 0.55	2.20 ± 0.84	1.60 ± 0.89
	C1	3.00 ± 0.00	3.00 ± 0.00	3.00 ± 0.00	2.40 ± 0.55	2.20 ± 0.84	1.80 ± 0.84
	C2	3.00 ± 0.00^a,b^	3.00 ± 0.00^c,d^	3.00 ± 0.00^e,f^	2.40 ± 0.55	2.20 ± 0.45^a,c,e^	2.00 ± 0.00^b,d,f^
Clarity of trabecular bone	Occipital	3.00 ± 0.00^a,b,c^	1.80 ± 0.45^a,d^	3.00 ± 0.00^d^	2.20 ± 0.45^b^	2.00 ± 0.71	1.80 ± 0.45^c^
	C1	2.60 ± 0.55	1.60 ± 0.89	2.80 ± 0.45^a,b,c^	1.60 ± 0.55^a^	1.60 ± 0.55^b^	1.20 ± 0.45^c^
	C2	3.00 ± 0.00^a,b,c,d^	1.60 ± 0.55^a,e^	3.00 ± 0.00^e,f,g,h^	2.00 ± 0.00^b,f^	2.00 ± 0.00^c,g^	1.80 ± 0.45^d,h^
Conspicuity of joint margin	Occipital	3.00 ± 0.00^a^	3.00 ± 0.00^b^	3.00 ± 0.00^c^	2.60 ± 0.55^d^	1.80 ± 0.45^a,b,c,d^	2.20 ± 0.84
	C1	3.00 ± 0.00^a^	3.00 ± 0.00^b^	3.00 ± 0.00^c^	2.40 ± 0.55	1.80 ± 0.84	1.80 ± 0.45^a,b,c^
	C2	3.00 ± 0.00^a,b,c^	3.00 ± 0.00^d,e,f^	3.00 ± 0.00^g,h,i^	2.00 ± 0.00^a,d,g^	1.80 ± 0.45^b,e,h^	1.60 ± 0.55^c,f,i^
Delineation of vertebral canal	C1	3.00 ± 0.00	3.00 ± 0.00	3.00 ± 0.00	2.60 ± 0.55	2.00 ± 0.71	2.40 ± 0.55
	C2	3.00 ± 0.00	3.00 ± 0.00	3.00 ± 0.00	2.60 ± 0.55	2.00 ± 0.71	2.40 ± 0.55

All data are presented as mean ± standard deviation.

^a-i^ Within a row, the same superscript indicated statistically significant differences between two groups using a Wilcoxon signed rank test. (significance level of *p*-value and< 0.05).

mFRACTURE, Fast field Echo Resembling a CT Using Restricted Echo-spacing- multiple echoes; sFRACTURE, Fast field Echo Resembling a CT Using Restricted Echo-spacing- single echo; PDW, Proton density-weighted; T2W, T2-weighted; and T1W, T1-weighted image; C1, the first cervical vertebrae; C2, the second vertebrae.

The mean and SD of the values obtained in the quantitative assessment are listed in Table 3. For all measured distances, geometrical agreement using Bland-Altman plot between MRI and CT was considered good to excellent, based on the limits of agreement (Table 4). The ICC results showed excellent agreement of > .8 for all measurements, except for the caudal height of the foramen magnum. In addition, among the values measured in the T2W images, all except the dens length showed a significant difference from those of CT. There were no significant differences between the distances measured using mFRACTURE, sFRACTURE, and T1W and those measured using CT.

**TABLE 3:** Overview of geometrical measurements by two observers for four anatomical distances. Measurement 1: Occipital bone length measured from the occipital protuberance to the ventral surface of the occipital bone (opisthion). Measurement 2: Caudal height of the foramen magnum measured from the inside of the basion to the inside of the opisthion. Measurement 3: Length of the dorsal arch of the atlas measured from the ventral line. Measurement 4: Maximum length of the dens measured from the ventral line.

Measurement	CT	mFRACTURE	sFRACTURE	PDW	T1W	T2W
**1**	21.23 ± 1.14^a^	20.97 ± 1.89	21.18 ± 1.95	21.96 ± 1.49	21.77 ± 1.47	22.55 ± 1.53^a^
**2**	14.74 ± 0.41^a,b^	14.72 ± 1.28	14.53 ± 1.04	13.78 ± 0.50a	14.08 ± 0.68	13.68 ± 0.89^b^
**3**	11.02 ± 1.16^a^	11.72 ± 0.87	11.53 ± 0.79	11.91 ± 0.79	11.±0.98	12.10 ± 0.68^a^
**4**	10.74 ± 1.16^a^	10.43 ± 1.80	10.51 ± 1.28	11.13 ± 1.39	10.65 ± 1.39	10.74 ± 1.29^a^

All data are presented as mean ± standard deviation.

^a, b^ Within a row, the same superscript indicated statistically significant differences between two groups using a Wilcoxon signed rank test. (significance level of *p*-value and< 0.05).

mFRACTURE, Fast field Echo Resembling a CT Using Restricted Echo-spacing- multiple echoes; sFRACTURE, Fast field Echo Resembling a CT Using Restricted Echo-spacing- single echo; PDW, Proton density-weighted; T2W, T2-weighted; and T1W, T1-weighted image; C1, the first cervical vertebrae; C2, the second vertebrae.

**TABLE 4:** Limits of agreement between CT of geometrical measurements for four anatomical distances. Measurement 1: Occipital bone length measured from the occipital protuberance to the ventral surface of the occipital bone (opisthion). Measurement 2: Caudal height of the foramen magnum measured from the inside of the basion to the inside of the opisthion. Measurement 3: Length of the dorsal arch of the atlas measured from the ventral line. Measurement 4: Maximum length of the dens measured from the ventral line.

Measurement	mFRACTURE	sFRACTURE	PDW	T1W	T2W
**1**	[-2.56; 2.05]	[-2.42; 2.32]	[-1.01; 2.47]	[-0.93; 2.01]	[-0.32; 2.96]
**2**	[-1.78; 1.74]	[-1.66; 1.24]	[-1.64; −0.28]	[-1.62; 0.30]	[-2.58; 0.47]
**3**	[-0.75; 2.14]	[-0.63; 1.66]	[0.00; 1.78]	[-0.44; 1.95]	[-0.36; 2.52]
**4**	[-1.68; 1.06]	[-0.75; 0.28]	[-0.37; 1.14]	[-0.91, 0.73]	[-0.66; 0.65]

mFRACTURE, Fast field Echo Resembling a CT Using Restricted Echo-spacing- multiple echoes; sFRACTURE, Fast field Echo Resembling a CT Using Restricted Echo-spacing- single echo; PDW, Proton density-weighted; T2W, T2-weighted; and T1W, T1-weighted image; C1, the first cervical vertebrae; C2, the second vertebrae.

**FIGURE 9:**
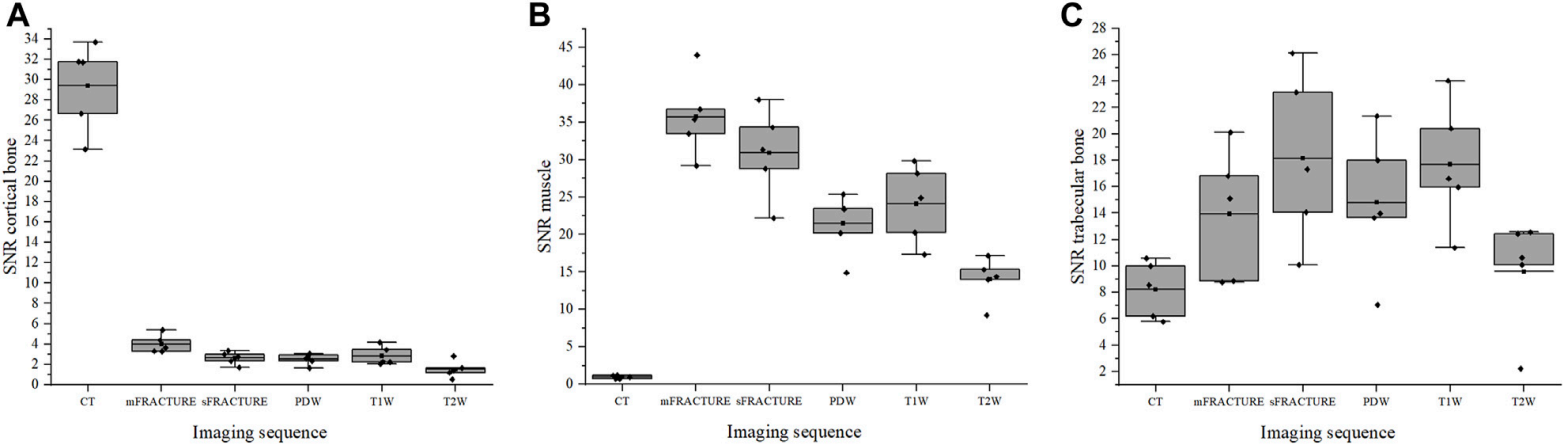
The signal-to-noise ratio (SNR) of cortical bone **(A)**, muscle **(B)**, and trabecular bone **(C)** for all evaluating methods (CT; computed tomography, mFRACTURE; fast field echo resembling CT using restricted echo-spacing- multiple echo, sFRACTURE; fast field echo resembling CT using restricted echo-spacing- single echo, PDW; proton density-weighted, T2W; T2-weighted, and T1W; T1-weighted images).

**FIGURE 10:**
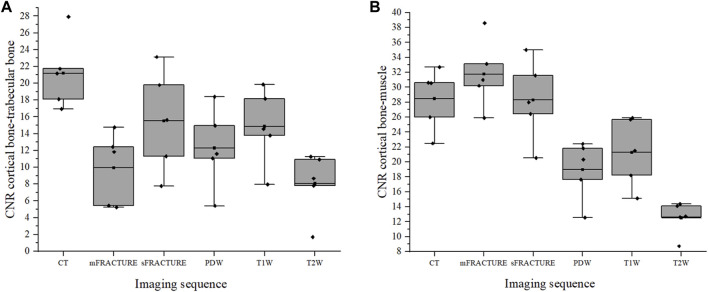
The contrast-to-noise ratio (CNR) of the cortical bone-trabecular bone **(A)** and cortical bone-muscle **(B)** for all evaluating methods. (CT; computed tomography, mFRACTURE; fast field echo resembling CT using restricted echo-spacing- multiple echo, sFRACTURE; fast field echo resembling CT using restricted echo-spacing- single echo, PDW; proton density-weighted, T2W; T2-weighted, and T1W; T1-weighted images).

The means and SDs of the SNRs and CNRs are shown in Table 5. The ICC results showed excellent agreement, with all >.8. The SNR of the cortical bone was higher with CT, showing a significant difference compared with all other MRI sequences with a mean of 29.40 (Figure 9A). By contrast, the muscle SNR was higher for MRI than for CT (Figure 9B). The SNR of the trabecular bone was higher with T1W, showing a significant difference compared with all other sequences except sFRACTURE, and sFRACTURE showing a significant difference compared with mFRACTURE (Figure 9C). The CNR of the cortical bone-trabecular bone was shown to have a low score, with both mFRACTURE and T2W averages below 10.0. No significant differences were found between the CT and MRI sequences, except for the mFRACTURE and T2W sequences, and a significant difference was observed between sFRACTURE and mFRACTURE sequences (Figure 10A). The CNR of the cortical bone-muscle showed no significant differences between CT, sFRACTURE, and mFRACTURE, and showed significant differences between CT and PDW, T1W, and T2W, with CT showing the highest contrast (Figure 10B).

**TABLE 5:** Comparison of magnetic resonance (MR) sequences and computed tomography (CT) for signal-to-noise ratio (SNR) and contrast-to-noise ratio (CNR).

Item	CT	mFRACTURE	sFRACTURE	PDW	T1W	T2W
SNR cortical bone	29.40 ± 4.35^a,b,c,d,e^	3.99 ± 0.89^a.f,g,h^	2.62 ± 0.65^b,f^	2.52 ± 0.56^c,g^	2.82 ± 0.92^d^	1.52 ± 0.83^e,h^
SNR muscle	0.93 ± 0.24^a,b,c,d,e^	35.74 ± 5.41^a,f,g,h^	30.92 ± 5.97^b,i,j,k^	21.48 ± 5.25^c,f,i,l,m^	24.10 ± 5.25^d,g,j,l,n^	14.02 ± 2.94^e,h,k,m,n^
SNR trabecular bone	8.22 ± 2.18^a^	13.93 ± 5.02^b,c^	18.14 ± 6.54^b^	14.81 ± 5.37^d,e^	17.68 ± 4.79^a,c,d,f^	9.58 ± 4.27^e,f^
CNR cortical bone-muscle	28.47 ± 4.15^a,b,c^	31.75 ± 4.64^d,e,f^	28.31 ± 5.47^g,h,i^	18.95 ± 4.03^a,d,g^	21.28 ± 4.69^b,e,h,j^	12.51 ± 2.26^c,f,i,j^
CNR cortical bone-trabecular bone	21.18 ± 4.27^a,b^	9.94 ± 4.34^a,c,d^	15.53 ± 6.21^c^	12.28 ± 4.84^e,f^	14.86 ± 4.61^d,e,g^	8.06 ± 3.85^b,f,g^

All data are presented as mean ± standard deviation.

^a-n^ Within a row, the same superscript indicated statistically significant differences between two groups using a Wilcoxon signed rank test. (significance level of *p*-value and< 0.05).

mFRACTURE, Fast field Echo Resembling a CT Using Restricted Echo-spacing- multiple echoes; sFRACTURE, Fast field Echo Resembling a CT Using Restricted Echo-spacing- single echo; PDW, Proton density-weighted; T2W, T2-weighted; and T1W, T1-weighted image.

## 4 Discussion

This study assessed the feasibility of evaluating the CCJ in dogs using the FRACTURE MRI sequence compared with CT and conventional MRI. Qualitative evaluation revealed that both the sFRACTURE and CT scores were significantly higher than those obtained with conventional MRI for cortical delineation and trabecular visibility. sFRACTURE and mFRACTURE scored highly for joint margin clarity and spinal canal clarity, as did the CT images. In the quantitative evaluation, distances measured on T2W images were significantly different from those measured on CT images, whereas there were no significant differences between the measurements taken using the other MRI sequences and those taken from CT images. T1W and sFRACTURE had higher SNRs than CT for trabecular bone. The CNR between the cortical bone and muscle was high for CT, sFRACTURE, and mFRACTURE, whereas that between the cortical bone and trabecular bone was low for mFRACTURE.

Because the dogs were scanned under anesthesia, there were very few motion artifacts to disturb the overall image quality, providing an optimal study environment for a thorough image quality comparison. Except in T1W, the other imaging types showed good image quality with an average score >2.0. In particular, the image quality obtained using mFRACTURE was high, and was significantly higher than that of PDW and T1W, showing that mFRACTURE is suitable for use for diagnostic purposes.

By contrast, sFRACTURE and CT had high scores for trabecular bone clarity, whereas mFRACTURE had low scores. This was attributed to the relatively narrow coverage of the trabecular bone in the cervical spine and high attenuation of the trabecular bone by the summation of MRI signals from five consecutive echo times in a sequence (Held et al., 2001; Deininger-Czermak et al., 2021). However, analysis of single- and multi-echo FRACTURE sequences together could facilitate a more comprehensive assessment, as mFRACTURE provides a higher CNR between the cortical and trabecular bones, joint margin, and cortical delineation, and a higher image quality than sFRACTURE. The acquisition time was 4 min 51 s for a single echo and 7 min 1 s for multiple echoes. Both sequences could be acquired faster than conventional 3D sequences. This result is in disagreement with human studies, in which FRACTURE sequences had a longer reconstruction time than conventional sequences because of the algebraic algorithm used to construct a CT-like image (Deininger-Czermak et al., 2021). However, in this study, sFRACTURE and mFRACTURE were obtained using 3D sequences with short acquisition times, and the combined time for both FRACTURE sequences was comparable to that of conventional sequences.

The conspicuity of the joint margins in the occipital bone was significantly lower on T2W than in all other sequence types. This could be due to the low image quality of T2W images or areas of high signal intensity on T2W, showing, for example, synovial fluid between the joint surfaces, which may be poorly visible on T2W, resulting in blurred boundaries that were difficult to assess.

In this study, we also found that the visualization of cortical boundaries was significantly better using sFRACTURE and mFRACTURE sequences, even though some conventional MRI images were assessable (Samim et al., 2019; Jans et al., 2021). Therefore, we concluded that FRACTURE helps assess cortical boundaries. In support of this, the CNR between the cortical bone and muscle was also higher in the FRACTURE sequences.

To the best of our knowledge, this is the first report of FRACTURE MRI of the CCJ in dogs. In humans, various types of MRI sequences such as UTE, ZTE, and bone MRI have been used to examine the skull, spine, shoulder, hip, and long bones (Bishop et al., 2013; Rathnayaka et al., 2013; Eley et al., 2014; Wiesinger et al., 2016; Stillwater et al., 2017; Breighner et al., 2019; de Mello et al., 2020; Stephen et al., 2021). Bone MRI, sCT, is a deep learning-based technique that performs 3D MRI-to-CT mapping to produce CT-like images (Florkow et al., 2020; Jans et al., 2021; Florkow et al., 2022). This technique has already been validated in the pelvis, sacroiliac joints, lumbar spine, cervical spine, and hip for radiotherapy and orthopedic and neurosurgical treatment planning by learning from paired MRI and CT data (Florkow et al., 2020; Jans et al., 2021; Morbée et al., 2021; Morbée et al., 2022). Based on the results of a previous human study comparing sCT and CT, a tendency to reduce fine details in the trabecular bone was observed in sCT, possibly because of the limited variety of images used to train the network, which would limit the identification of certain pathologies, such as intravertebral bone attachments. In contrast, the sFRACTURE sequence in our study showed that the trabecular bone could be visualized at a level similar to that on CT.

Conventional MRI sequences have an echo time (TE) of more than 1 ms, which results in little or no detectable signal from the cortical bone. However, nominal TEs of less than 200 μs can be achieved using half-synchronized radio frequency (RF) pulses or short hard pulses, radial or spiral mapping of K-space, and other techniques. The UTE pulse sequence uses short RF pulses for signal excitation and collects data as the readout gradient increases (TE 8–50 µs). Consequently, UTE pulse sequences can detect signals in the cortical bone and directly image and quantify T1, T2*, and water concentrations in bone tissue. In humans, several studies have evaluated the ability of CLBC 3D UTE to characterize and delineate bone structures and have reported its strong performance in shoulder and cervical spine imaging (Chang et al., 2015; Larson et al., 2016; Deininger-Czermak et al., 2021). The qualitative scores for UTE showed more similarity to CT than to FRACTURE, and there was no difference in the geometric measurements between UTE and FRACTURE(25). However, the hardware and software requirements for UTE and ZTE acquisition include fast transmission and receive switching, precise RF waveform transmission, high-gradient performance, tilt compensation, and no commercially available pulse sequences, which limit the number of MRI scanners capable of performing UTE and ZTE acquisition in practice.

In this study, as in previous ones (Rathnayaka et al., 2013; Wiesinger et al., 2016), most lengths measured using FRACTURE were not significantly different from those measured on CT. Regarding the quantitative assessment of all modalities, CT and FRACTURE were able to measure lengths in as similar a cross section as possible, as CT and FRACTURE are 3D techniques that use isotropic voxels, allowing for accurate midsagittal plane correction. However, the lengths measured on T2W images were significantly different from those measured on CT images. This suggests that T2W may not be accurate enough to measure the distance between bone margins on MRI images. This may also suggest that additional visual information about the surrounding soft tissue and fluid structures influences the assessment of distance on MRI in humans. The occipital bone length, length of the dorsal arch of the atlas, and maximum length of the dens showed excellent ICC agreement, and the caudal height of the foramen magnum showed poor ICC agreement, which may be due to the small number of patients; however, it is possible that the length of the foramen magnum is more affected by posture than we expected.

In veterinary medicine, various studies have used CT and MRI to diagnose abnormalities of the CCJ in dogs (Lu et al., 2003; García-Real et al., 2004; Cerda-Gonzalez et al., 2009a; Cerda-Gonzalez et al., 2009b; Carrera et al., 2009; Schmidt et al., 2009; Parry et al., 2010; Marino et al., 2012; Stalin et al., 2015; Kiviranta et al., 2017; Planchamp et al., 2022). CT has been used mainly for morphologic evaluation of the bones. One study evaluated the morphological variation between dogs with and without atlantoaxial subluxation using CT (Parry et al., 2010). Two studies assessed the shape and volume of the cranial cavity and caudal fossa using CT (García-Real et al., 2004; Schmidt et al., 2009). MRI studies have primarily been used to determine the presence of spinal cord pathology and disorders of cerebrospinal fluid flow (Lu et al., 2003; Cerda-Gonzalez et al., 2009b). However, because of the limitations of using MRI alone to assess osseous structures, a number of studies have combined both CT and MRI (Cerda-Gonzalez et al., 2009a; Marino et al., 2012; Stalin et al., 2015; Kiviranta et al., 2017; Planchamp et al., 2022). In one study of atlanto-occipital overlapping, CT was used to assess the position of the atlas and occipital bones, and MRI was used to assess medullary kinking, compression of the cerebellum, and ventriculomegaly (Cerda-Gonzalez et al., 2009a). The combination of the conventional MRI with the FRACTURE sequence, which is useful for evaluating osseous structures, will allow MRI to be used to evaluate bone morphology and nerve injury at the one time, reducing complexity.

Our study has some limitations. First, in post-processing, inverting the greyscale makes areas that were identified as dark with low signal in the MRI image appear brighter. Therefore, areas such as air, which are not bones, may appear bright when inverted due to lack of signal, so when viewing FRACTURE images, it is important to refer to anatomical structures and other MRI sequences to avoid misinterpretation and consult a radiologist if necessary. Second, all examinations were performed on a small number of healthy dogs of the same body size. Therefore, the images may not be the same as those of diseased dogs. Further studies on the application of FRACTURE MRI in large populations of healthy dogs of various body sizes and dogs with craniocervical diseases are necessary. Furthermore, there was no comparison between FRACTURE and other MRI sequences that provide CLBC images; therefore, it is difficult to determine the differences between the imaging types.

Despite these limitations, the FRACTURE MRI sequences showed higher cortical delineation and trabecular bone visibility than T2-weighted, T1-weighted, and PDW images of the CCJ, as did CT. The CNR between the cortical bone and muscle was high, similar to that on CT, and sFRACTURE showed a high SNR of the trabecular bone and a high CNR between the cortical and trabecular bone. In conclusion, FRACTURE sequences can be used as an adjunct to conventional MR sequences for evaluating CCJ bones in dogs (Weiger et al., 2013; Weiger et al., 2011).

## Data Availability

The original contributions presented in the study are included in the article/Supplementary Material, further inquiries can be directed to the corresponding author.
