# Supplementary material for: Fast field echo resembling CT using restricted echo-spacing (FRACTURE) MR sequence can provide craniocervical region images comparable to a CT in dogs
Source: Front Bioeng Biotechnol. 2024 Feb 27;12:1297675. doi: 10.3389/fbioe.2024.1297675 (PMC10927716; doi:10.3389/fbioe.2024.1297675)
Supplement: Supplementary file 1 [file DataSheet1.docx]

Supplementary Material

# Supplementary Figures and Tables

## Supplementary Figures


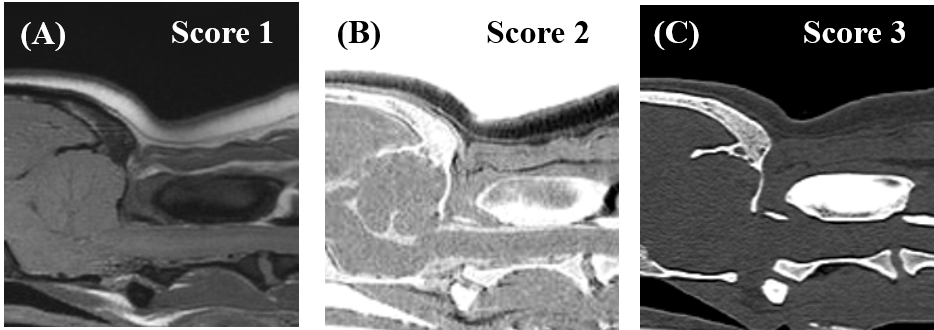


**Figure 1.** Qualitative evaluation of the image qualities on T1-weighted (A), single echo-FRACTURE (B), CT (C) images of craniocervical region in a beagle. 1 = Artifacts present, affecting the images; 2 = Artifacts were present but minimal, not affecting the images; and 3 = Artifacts were absent.


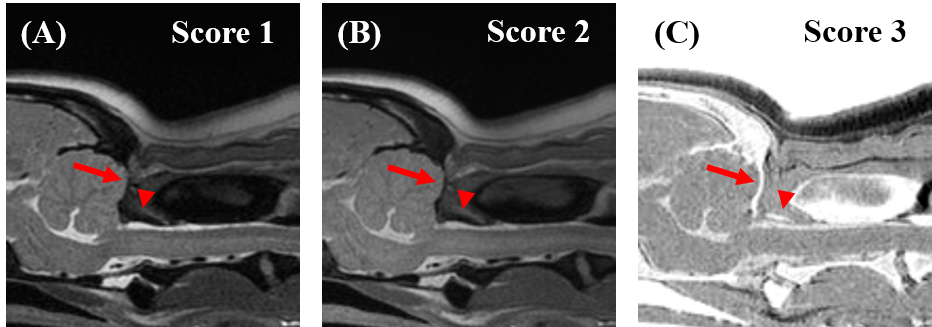


**Figure 2.** Qualitative evaluation of the visuality of the cortical delineation of occipital region (arrow) and dorsal arch of atlas (arrow head) on T2-weighted (A), Proton density-weighted (B), single echo-FRACTURE (C) images of craniocervical region in a beagle. 1 = The border of the cortical bone was too blurry to be distinguished from the surrounding soft tissue; 2 = Most cortical bones were well distinguished, but there were some blurry parts; 3 = The cortical bone was clearly distinguishable from the surrounding soft tissue.


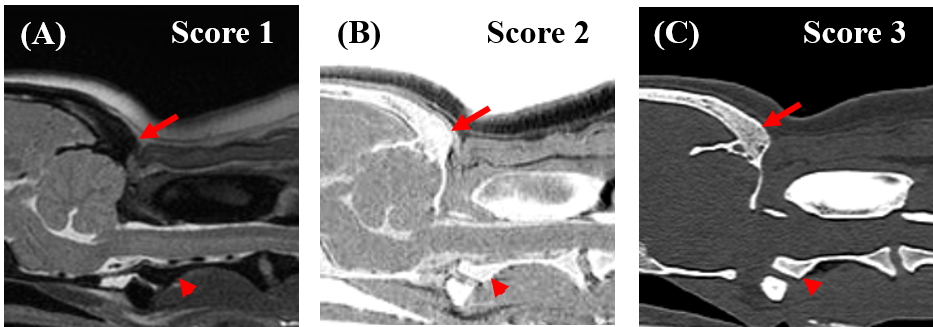


**Figure 3.** Qualitative evaluation of the clarity of trabecular bone of occipital region (arrow) and axis (arrow head) on T2-weighted (A), single echo-FRACTURE (B), CT (C) images of craniocervical region in a beagle. 1 = The trabecular bone appeared homogeneous overall, and there were no visible trabecular patterns; 2 = The trabecular pattern was visible but not clear; 3 = The trabecular and cortical bones were well distinguished, and the trabecular pattern was visible.


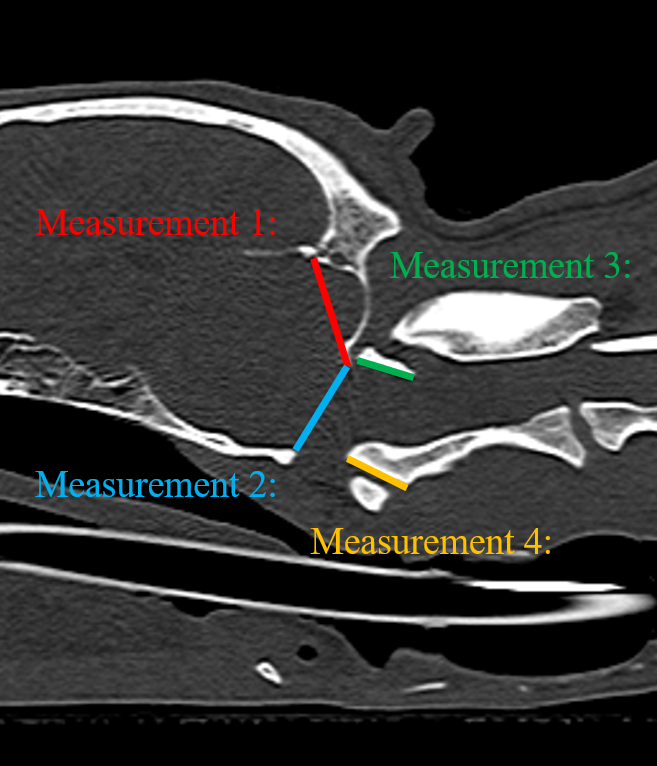


**Figure 4.** Illustration of measurements 1–4 to evaluate geometric accuracy on CT sagittal images. Measurement 1: Occipital bone length measured from the occipital protuberance to the ventral surface of the occipital bone (opisthion). Measurement 2: Caudal height of the foramen magnum measured from the inside of the basion to the inside of the opisthion. Measurement 3: Length of the dorsal arch of the atlas measured from the ventral line. Measurement 4: Maximum length of the dens measured from the ventral line.


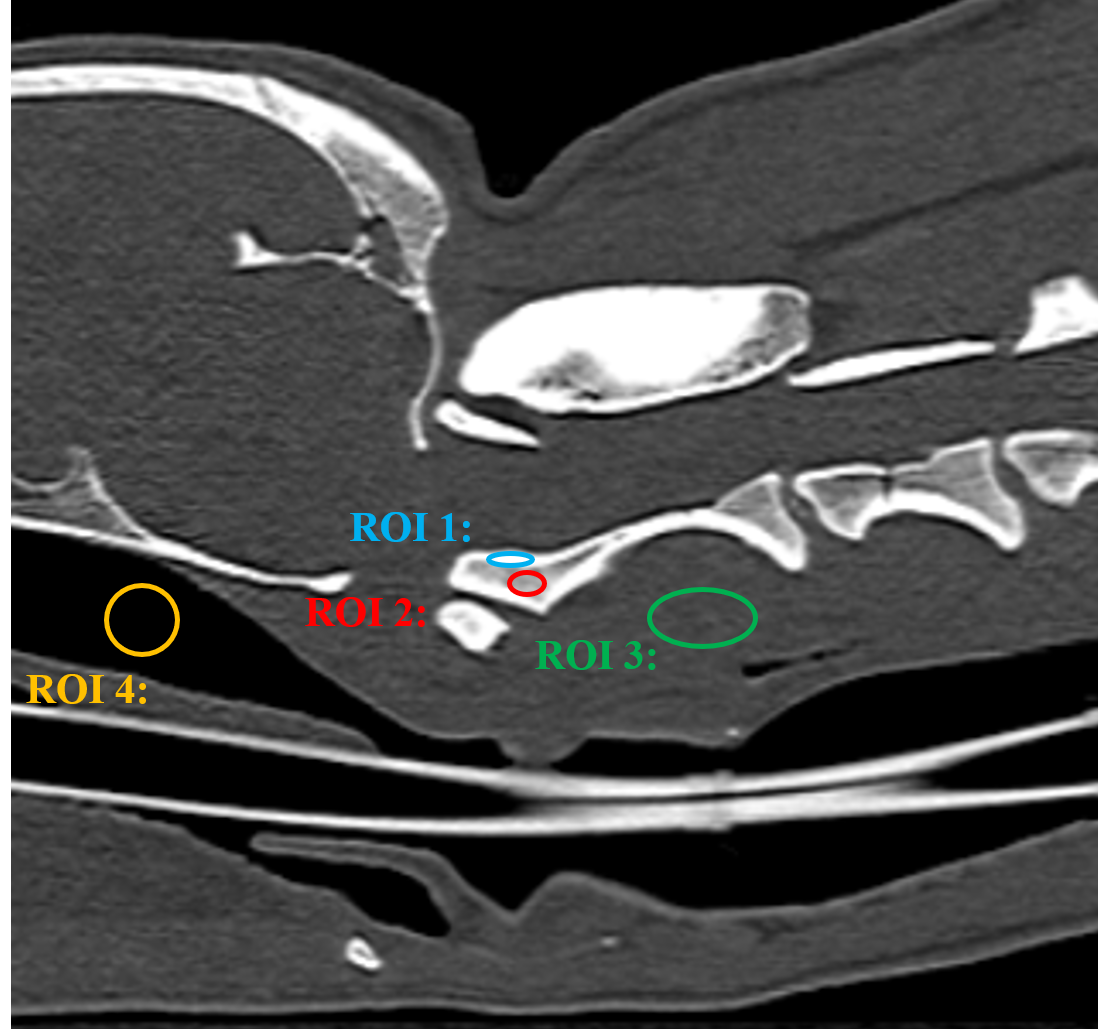


**Figure 5.** CT image of a craniocervical region in the sagittal plane. The signal intensity of the cortical bone was measured by placing a oval region of interest (ROI) over the cortical bone of axis (ROI 1). The signal intensity of the trabecular bone was measured by placing a oval region of interest (ROI) over the trabecular bone of axis (ROI 2). The signal intensity of the surrounding muscle was measured by placing a circular or oval ROI over the muscle adjacent to the axis (ROI 3). Background noise was measured by placing a circular or oval ROI in the air of the nasopharynx (ROI 4).


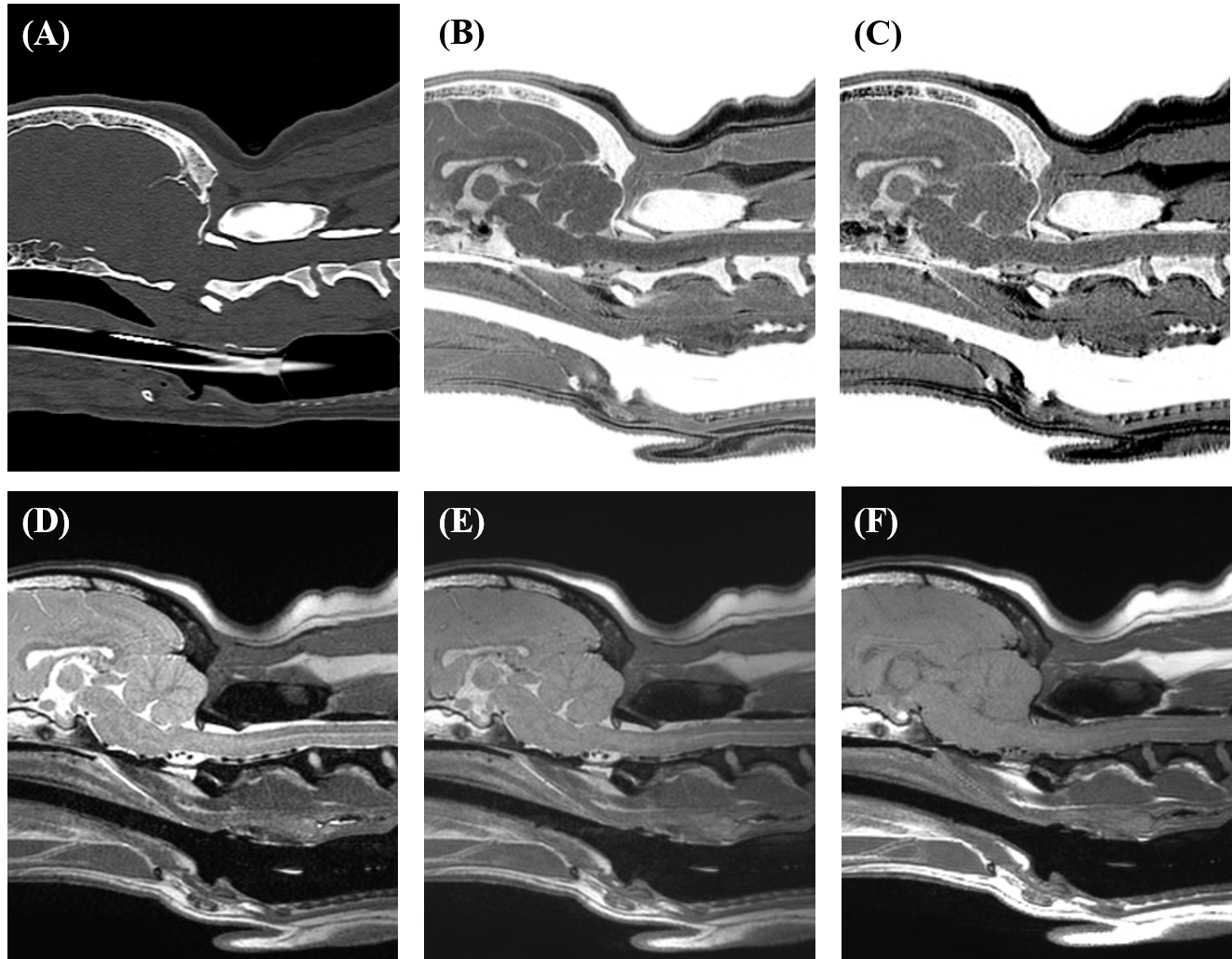


**Figure 6.** CT (A) and MR (B-F) images of the craniocervical junction (CCJ) in a beagle. Multiple echo-FRACTURE (B) and single echo-FRACTURE (C) showed higher cortical delineation and trabecular bone visibility than T2-weighted (D), proton density-weighted (E), and T1-weighted (F) images of CCJ, similar to that observed on CT images.


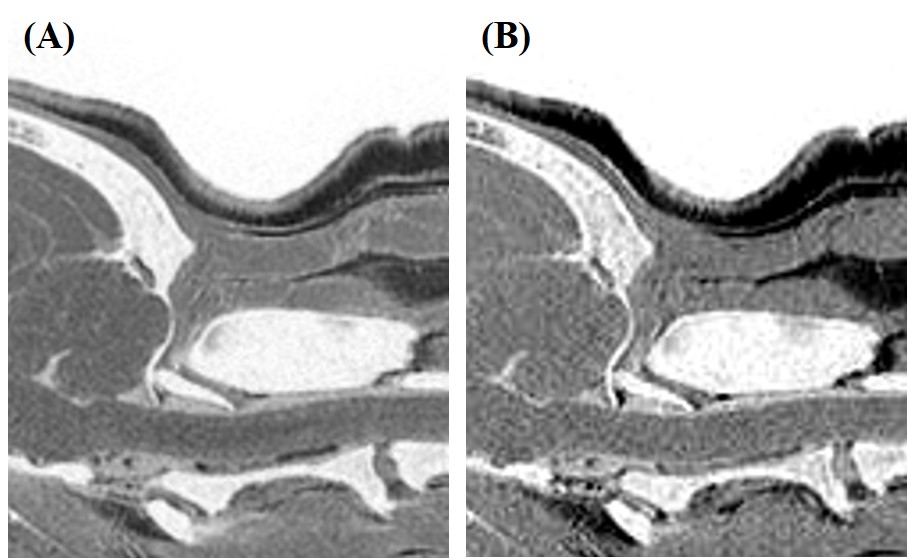


**Figure 7.** Multiple echo-FRACTURE (A) and single echo-FRACTURE (B) images of the craniocervical junction (CCJ) in a beagle. Single echo-FRACTURE showed higher trabecular bone visibility than multiple echo-FRACTURE.


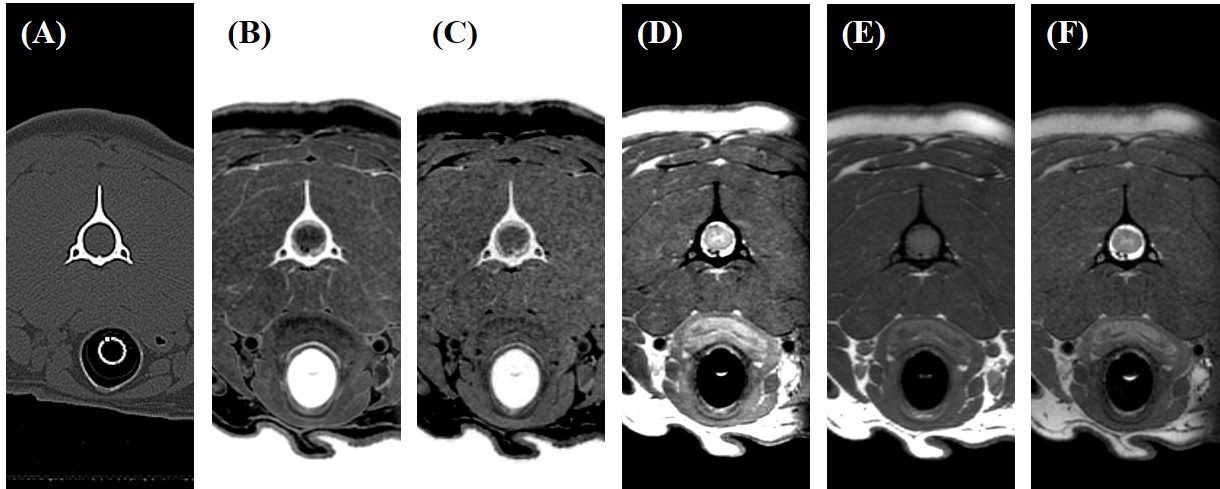


**Figure 8.** CT (A) and MR (B-F) transverse images of the vertebral canal at C2 (axis) in a beagle. All sequences (CT, multiple echo-FRACTURE (B), single echo-FRACTURE (C), proton density-weighted (D), T1-weighted (E), and T2-weighted (F)) had high scores for the evaluation of vertebral canal delineation.


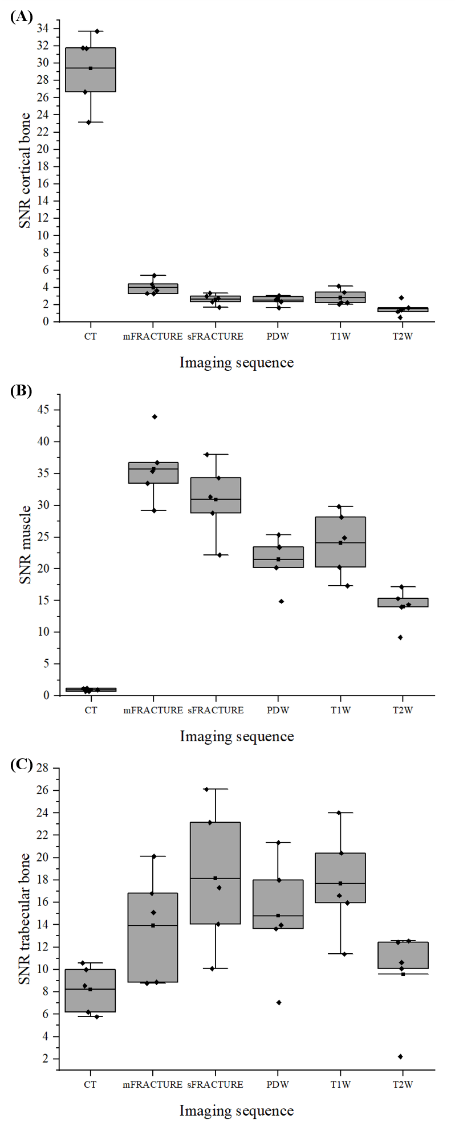


**Figure 9.** The signal-to-noise ratio (SNR) of the cortical bone (A), muscle (B), and trabecular bone (C) for all evaluating methods. (CT; computed tomography, mFRACTURE; fast field echo resembling CT using restricted echo-spacing- multiple echo, sFRACTURE; fast field echo resembling CT using restricted echo-spacing- single echo, PDW; proton density-weighted, T2W; T2-weighted, and T1W; T1-weighted images)


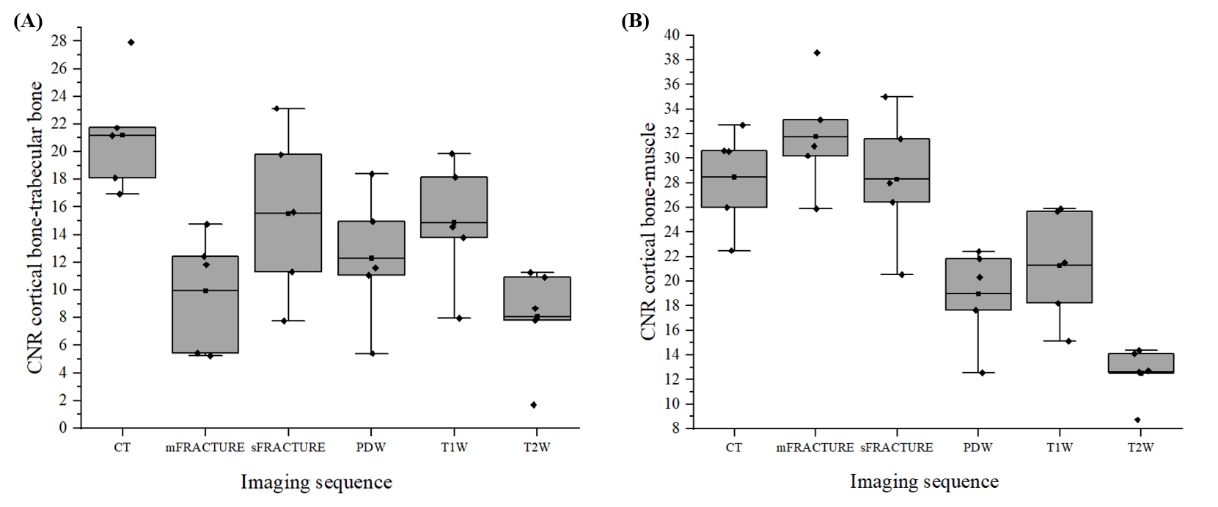


**Figure 10.** The contrast-to-noise ratio (CNR) of the cortical bone-trabecular bone (A) and cortical bone-muscle (B) for all evaluating methods. (CT; computed tomography, mFRACTURE; fast field echo resembling CT using restricted echo-spacing- multiple echo, sFRACTURE; fast field echo resembling CT using restricted echo-spacing- single echo, PDW; proton density-weighted, T2W; T2-weighted, and T1W; T1-weighted images)

**Table 1****.** MRI parameters and sequences.

| **Parameter** | **MR sequences** | | | | |
| --- | --- | --- | --- | --- | --- |
|  | **3D FRACTURE**  **Multi-echo** | **3D FRACTURE**  **Single-echo** | **3D PDW** | **3D T1W** | **3D T2W** |
| **Number of In phase echo** | 4 | 1 |  |  |  |
| **TR (ms)** | 16 | 5.4 | 1100 | 350 | 1500 |
| **TE (ms)** | In phase (2.3)  Echo-spacing (2.3) | In phase (2.3) | 34 | 19 | 100 |
| **Flip angle** | 12 | 8 | 90 | 90 | 90 |
| **Slice interval** | 0.6 | 0.6 | 0.6 | 0.6 | 0.6 |
| **FOV (mm)** | 160 x 160 x 60 | 160 x 160 x 60 | 160 x 160 x 60 | 160 x 160 x 60 | 160 x 160 x 60 |
| **Matrix** | 268 x 266 | 268 x 266 | 268 x 266 | 268 x 266 | 268 x 235 |
| **Reconstruction Matrix** | 320 | 288 | 320 | 320 | 320 |
| **NEX** | 1 | 1 | 1 | 1 | 1 |
| **Acquisition time(min:sec)** | 7:01 | 4:51 | 12:57 | 11:55 | 13:31 |

MR; Magnetic resonance image, CT; computed tomography, mFRACTURE; Fast field Echo Resembling a CT Using Restricted Echo-spacing- multiple echoes, sFRACTURE; Fast field Echo Resembling a CT Using Restricted Echo-spacing- single echo, PDW; Proton density-weighted, T2W;T2-weighted, and T1W; T1-weighted image, TR; Repeated time, TE; Echo time, FOV; field of view, NEX; number of excitation

**Table 2.** Scores of several parameters of magnetic resonance imaging (MRI) and computed tomography (CT).

| **Item** | **Level** | **CT** | | **mFRACTURE** | | | **sFRACTURE** | | | | **PDW** | | | **T1W** | | | **T2W** |
| --- | --- | --- | --- | --- | --- | --- | --- | --- | --- | --- | --- | --- | --- | --- | --- | --- | --- |
| **Image quality and artifacts** |  | 2.80±0.45^a^ | | | 2.80±0.45^b, c^ | | | 2.20±0.45^d^ | | 2.00±0.71^b, e^ | | | 1.00±00^a, c, d, f^ | | | 2.20±0.45^e, f^ | |
| **Visualization of cortical delineation** | Occipital  C1  C2 | 3.00±0.00  3.00±0.00  3.00±0.00^a,b^ | | | 3.00±0.00  3.00±0.00  3.00±0.00^c,d^ | | | 3.00±0.00  3.00±0.00  3.00±0.00^e,f^ | | 2.60±0.55  2.40±0.55  2.40±0.55 | | | 2.20±0.84  2.20±0.84  2.20±0.45^a,c,e^ | | | 1.60±0.89  1.80±0.84  2.00±0.00^b,d,f^ | |
| **Clarity of trabecular bone** | Occipital  C1  C2 | 3.00±0.00^a,b,c^  2.60±0.55  3.00±0.00 ^a,b,c,d^ | | | 1.80±0.45^a,d^  1.60±0.89  1.60±0.55^a,e^ | | | 3.00±0.00^d^  2.80±0.45^a,b,c^  3.00±0.00^e,f,g,h^ | | 2.20±0.45^b^  1.60±0.55^a^  2.00±0.00^b,f^ | | | 2.00±0.71  1.60±0.55^b^  2.00±0.00^c,g^ | | | 1.80±0.45^c^  1.20±0.45^c^  1.80±0.45^d,h^ | |
| **Conspicuity of joint margin** | Occipital  C1  C2 | 3.00±0.00^a^  3.00±0.00^a^  3.00±0.00^a,b,c^ | 3.00±0.00^b^  3.00±0.00^b^  3.00±0.00^d,e,f^ | | | 3.00±0.00^c^  3.00±0.00^c^  3.00±0.00^g,h,i^ | | | 2.60±0.55^d^  2.40±0.55  2.00±0.00^a,d,g^ | | | 1.80±0.45^a,b,c,d^  1.80±0.84  1.80±0.45^b,e,h^ | | | 2.20±0.84  1.80±0.45^a,b,c^  1.60±0.55^c,f,i^ | | |
| **Delineation of vertebral canal** | C1  C2 | 3.00±0.00  3.00±0.00 | 3.00±0.00  3.00±0.00 | | | 3.00±0.00  3.00±0.00 | | | 2.60±0.55  2.60±0.55 | | | 2.00±0.71  2.00±0.71 | | | 2.40±0.55  2.40±0.55 | | |

All data are presented as mean ± standard deviation.

^a - i^ Within a row, the same superscript indicated statistically significant differences between two groups using a Wilcoxon signed rank test. (significance level of P-value < 0.05).

mFRACTURE; Fast field Echo Resembling a CT Using Restricted Echo-spacing- multiple echoes, sFRACTURE; Fast field Echo Resembling a CT Using Restricted Echo-spacing- single echo, PDW; Proton density-weighted, T2W;T2-weighted, and T1W; T1-weighted image, C1; the first cervical vertebrae, C2; the second vertebrae.

**Table 3.** Overview of geometrical measurements for four anatomical distances.

| **Measurement** | **CT**  (mm) | **mFRACTURE**  (mm) | **sFRACTURE**  (mm) | **PDW**  (mm) | **T1W**  (mm) | **T2W**  (mm) |
| --- | --- | --- | --- | --- | --- | --- |
| **1** | 21.23±1.14^a^ | 20.97±1.89 | 21.18±1.95 | 21.96±1.49 | 21.77±1.47 | 22.55±1.53^a^ |
| **2** | 14.74±0.41^a,b^ | 14.72±1.28 | 14.53±1.04 | 13.78±0.50^a^ | 14.08±0.68 | 13.68±0.89^b^ |
| **3** | 11.02±1.16^a^ | 11.72±0.87 | 11.53±0.79 | 11.91±0.79 | 11.±0.98 | 12.10±0.68^a^ |
| **4** | 10.74±1.16^a^ | 10.43±1.80 | 10.51±1.28 | 11.13±1.39 | 10.65±1.39 | 10.74±1.29^a^ |

All data are presented as mean ± standard deviation.

^a - b^ Within a row, the same superscript indicated statistically significant differences between two groups using a Wilcoxon signed rank test. (significance level of P-value < 0.05).

mFRACTURE; Fast field Echo Resembling a CT Using Restricted Echo-spacing- multiple echoes, sFRACTURE; Fast field Echo Resembling a CT Using Restricted Echo-spacing- single echo, PDW; Proton density-weighted, T2W;T2-weighted, and T1W; T1-weighted image, C1; the first cervical vertebrae, C2; the second vertebrae.

Measurement 1: Occipital bone length measured from the occipital protuberance to the ventral surface of the occipital bone (opisthion). Measurement 2: Caudal height of the foramen magnum measured from the inside of the basion to the inside of the opisthion. Measurement 3: Length of the dorsal arch of the atlas measured from the ventral line. Measurement 4: Maximum length of the dens measured from the ventral line.

**Table 4.** Limits of agreement between CT of geometrical measurements for four anatomical distances.

| **Measurement** | **mFRACTURE**  (mm) | **sFRACTURE**  (mm) | **PDW**  (mm) | **T1W**  (mm) | **T2W**  (mm) |
| --- | --- | --- | --- | --- | --- |
| **1** | [-2.56; 2.05] | [-2.42; 2.32] | [-1.01; 2.47] | [-0.93; 2.01] | [-0.32; 2.96] |
| **2** | [-1.78; 1.74] | [-1.66; 1.24] | [-1.64; -0.28] | [-1.62; 0.30] | [-2.58; 0.47] |
| **3** | [-0.75; 2.14] | [-0.63; 1.66] | [0.00; 1.78] | [-0.44; 1.95] | [-0.36; 2.52] |
| **4** | [-1.68; 1.06] | [-0.75; 0.28] | [-0.37; 1.14] | [-0.91, 0.73] | [-0.66; 0.65] |

mFRACTURE; Fast field Echo Resembling a CT Using Restricted Echo-spacing- multiple echoes, sFRACTURE; Fast field Echo Resembling a CT Using Restricted Echo-spacing- single echo, PDW; Proton density-weighted, T2W;T2-weighted, and T1W; T1-weighted image, C1; the first cervical vertebrae, C2; the second vertebrae.

Measurement 1: Occipital bone length measured from the occipital protuberance to the ventral surface of the occipital bone (opisthion). Measurement 2: Caudal height of the foramen magnum measured from the inside of the basion to the inside of the opisthion. Measurement 3: Length of the dorsal arch of the atlas measured from the ventral line. Measurement 4: Maximum length of the dens measured from the ventral line.

**Table 5.** Comparison of magnetic resonance (MR) sequences and computed tomography (CT) for signal-to-noise ratio (SNR) and contrast-to-noise ratio (CNR).

| **Item** | **CT** | **mFRACTURE** | **sFRACTURE** | **PDW** | **T1W** | **T2W** |
| --- | --- | --- | --- | --- | --- | --- |
| **SNR cortical bone** | 29.40±4.35^a,b,c,d,e^ | 3.99±0.89^a.f,g,h^ | 2.62±0.65^b,f^ | 2.52±0.56^c,g^ | 2.82±0.92^d^ | 1.52±0.83^e,h^ |
| **SNR muscle** | 0.93±0.24^a,b,c,d,e^ | 35.74±5.41^a,f,g,h^ | 30.92±5.97^b,i,j,k^ | 21.48±5.25^c,f,i,l,m^ | 24.10±5.25^d,g,j,l,n^ | 14.02±2.94^e,h,k,m,n^ |
| **SNR trabecular bone** | 8.22±2.18^a^ | 13.93±5.02^b,c^ | 18.14±6.54^b^ | 14.81±5.37^d,e^ | 17.68±4.79^a,c,d,f^ | 9.58±4.27^e,f^ |
| **CNR cortical bone-muscle** | 28.47±4.15^a,b,c^ | 31.75±4.64^d,e,f^ | 28.31±5.47^g,h,i^ | 18.95±4.03^a,d,g^ | 21.28±4.69^b,e,h,j^ | 12.51±2.26^c,f,i,j^ |
| **CNR cortical bone-trabecular bone** | 21.18±4.27^a,b^ | 9.94±4.34^a,c,d^ | 15.53±6.21^c^ | 12.28±4.84^e,f^ | 14.86±4.61^d,e,g^ | 8.06±3.85^b,f,g^ |

All data are presented as mean ± standard deviation.

^a - n^ Within a row, the same superscript indicated statistically significant differences between two groups using a Wilcoxon signed rank test. (significance level of P-value < 0.05).

mFRACTURE; Fast field Echo Resembling a CT Using Restricted Echo-spacing- multiple echoes, sFRACTURE; Fast field Echo Resembling a CT Using Restricted Echo-spacing- single echo, PDW; Proton density-weighted, T2W;T2-weighted, and T1W; T1-weighted image
